# Supplementary material for: Novel Antimicrobial Peptides Designed Using a Recurrent Neural Network Reduce Mortality in Experimental Sepsis
Source: Antibiotics (Basel). 2022 Mar 18;11(3):411. doi: 10.3390/antibiotics11030411 (PMC8944797; doi:10.3390/antibiotics11030411)

Table S1. MIC values calculations against *K. aerogenes.*

isolate #	PEP-38 — 1st MIC	PEP-38 — 2nd MIC	PEP-38 — 3rd MIC	Median PEP-38 MIC		PEP-138 — 1st MIC	PEP-138— 2nd MIC	PEP-138 — 3rd MIC	Median PEP-138 MIC
1	16	16	8	16		2	2	1	2
2	2	4	4	4		0,5	0,5	1	0,5
3	8	16	8	8		2	1	1	1
4	4	4	2	4		0,5	1	1	1
5	16	8	16	16		1	1	2	1
6	8	8	8	8		2	4	4	4
7	2	2	4	2		2	4	2	2
8	8	8	8	8		2	2	2	2
9	8	4	4	4		2	2	2	2
10	8	8	8	8		4	4	2	4
11	4	2	4	4		0,5	2	2	2
12	4	4	4	4		4	8	4	4
Median				6					2
First quartile (25)				4					1
Third quartile (75)				8					2,5

Table S2. MIC values calculations against *K. pneumoniae.*

isolate #	PEP-38 — 1st MIC	PEP-38 — 2nd MIC	PEP-38 — 3rd MIC	Median PEP-38 MIC		PEP-138 — 1st MIC	PEP-138— 2nd MIC	PEP-138 — 3rd MIC	Median PEP-138 MIC
1	8	32	16	16		4	2	4	4
2	8	8	8	8		2	1	1	1
3	4	8	8	8		1	1	1	1
4	2	4	4	4		1	1	1	1
5	4	4	4	4		0,5	1	1	1
6	4	4	4	4		2	1	1	1
7	4	4	4	4		4	4	2	4
8	4	4	8	4		4	4	4	4
9	8	8	8	8		2	2	2	2
10	16	32	32	32		8	4	8	8

11	32	32	32	32		0,5	0,5	0,5	0,5
12	16	8	8	8		1	2	1	1
13	0,5	0,5	0,5	0,5		0,25	0,25	0,5	0,25
14	2	2	8	2		1	4	4	4
15	8	8	8	8		2	2	2	2
16	4	4	4	4		4	2	2	2
17	8	8	4	8		2	2	2	2
18	16	32	16	16		2	4	4	4
Median				8					2
First quartile (25)				4					1
Third quartile (75)				8					4

Table S3. MIC values calculations against *P. aeruginosa.*

isolate #	PEP-38 — 1st MIC	PEP-38 — 2nd MIC	PEP-38 — 3rd MIC	Median PEP-38 MIC		PEP-138 — 1st MIC	PEP-138— 2nd MIC	PEP-138 — 3rd MIC	Median PEP-138 MIC
1	R	R	R	R		4	4	4	4
2	R	R	R	R		2	2	4	2
3	R	R	R	R		2	4	4	4
4	R	R	R	R		2	4	4	4
5	R	R	R	R		2	2	4	2
6	R	R	R	R		4	4	2	4
7	R	R	R	R		2	2	2	2
8	R	R	R	R		0,5	1	1	1
9	R	R	R	R		4	2	2	2
10	R	R	R	R		2	2	2	2
11	R	R	R	R		2	2	2	2
12	R	R	R	R		1	2	4	2
13	R	R	R	R		2	2	2	2
14	R	R	R	R		2	2	1	2
15	R	R	R	R		1	2	4	2
16	R	R	R	R		4	4	2	4
17	R	R	R	R		4	4	4	4
Median									2

First quartile (25)									2
Third quartile (75)									4

[a] R – resistant to 32 µg/ml of PEP-38.

Table S4. Survival proportions (%) in all experimental animal groups.

Days after infecting	Control (n=12)	PEP-36 (n=12)	PEP-38 (n=12)	PEP-137 (n=12)
0	100	100	100	100
1	66,7	91,7	66,7	91,7
2	16,7	75	41,7	66,7
3	0	66,7	8,3	50
5		66,7	8,3	50

Figure S1. Probability of survival: control vs PEP-36.

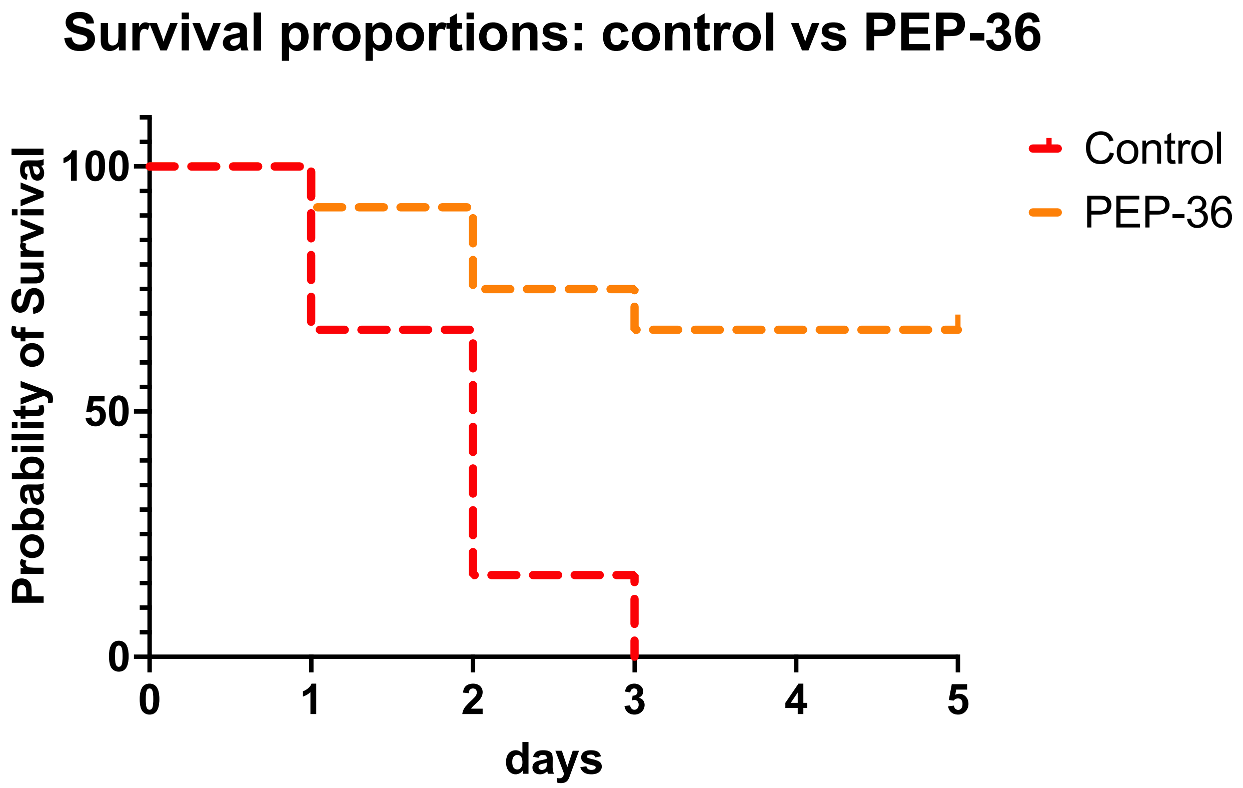

Figure S2. Probability of survival: control vs PEP-38.

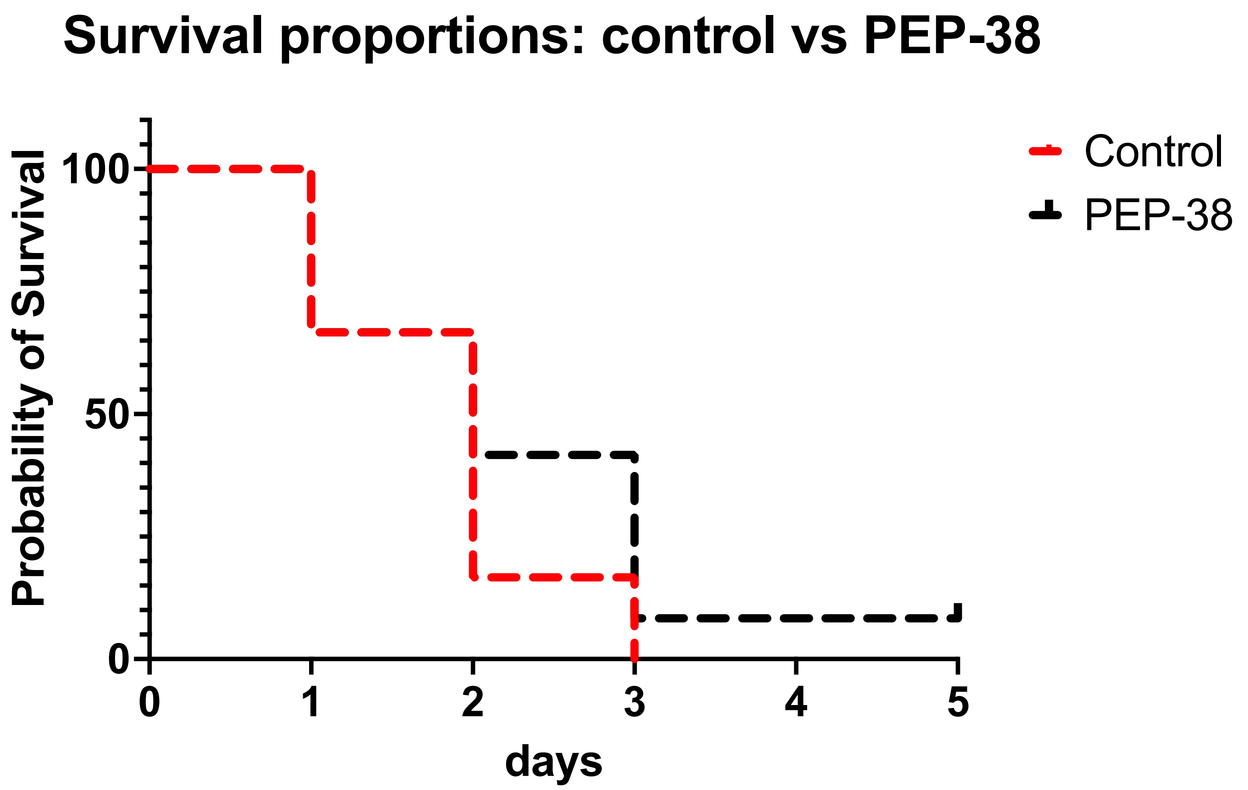

Figure S3. Probability of survival: control vs PEP-137.

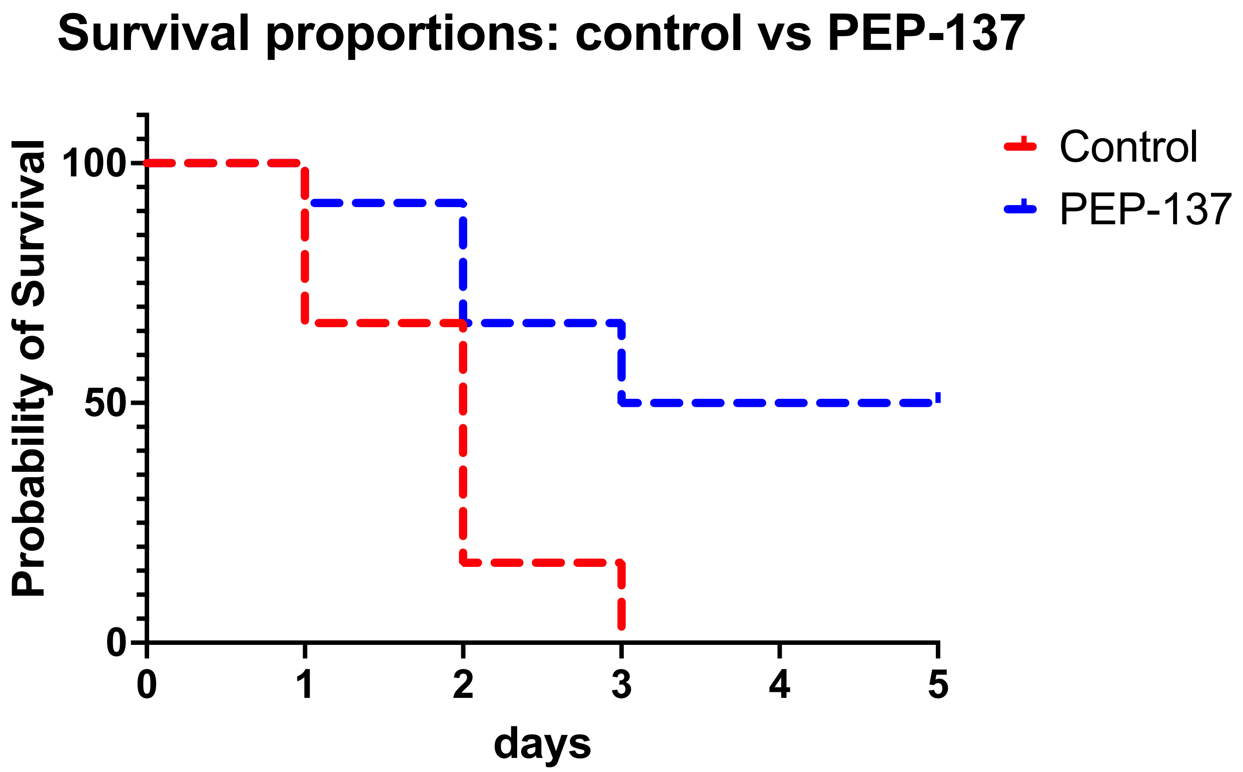

Figure S4. Time evolution of root mean squared deviation (RMSD) of peptides (A) and membrane (B) throughout the MD simulation.

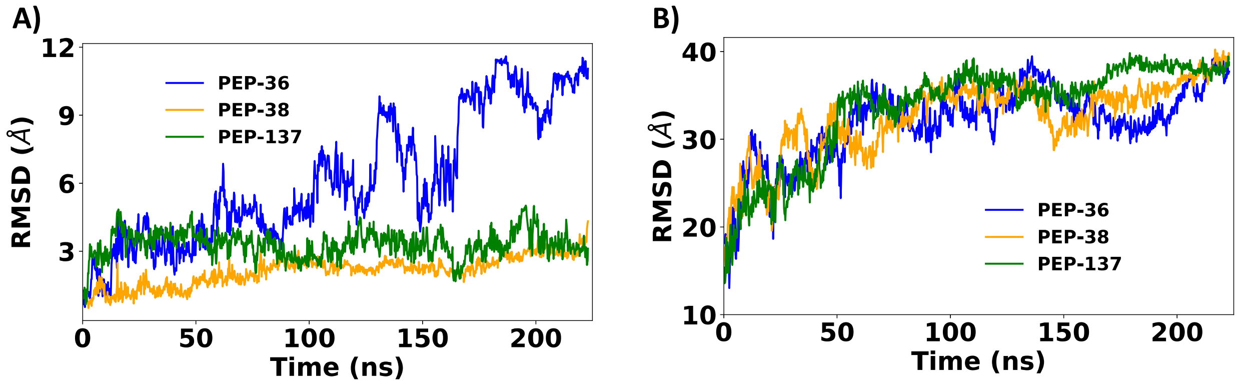

Figure S5. Percentage of hydrogen bonding between peptide residues and membrane averaged over the last 50ns. Color scheme: hydrogen bonds with backbone CO (red), backbone NH (blue), and sidechain (SC) (green).

To identify the important amino acids crucial for interaction with bacterial membrane, we calculated the percentage of hydrogen bonding between peptide residues and membrane averaged over the last 50ns and is plotted in Figure S5. It was shown that for all three peptides the side chain (green bar) of amino acids are main contributor to the h-bond with the lipid molecule. Most of these amino acids are lysine.

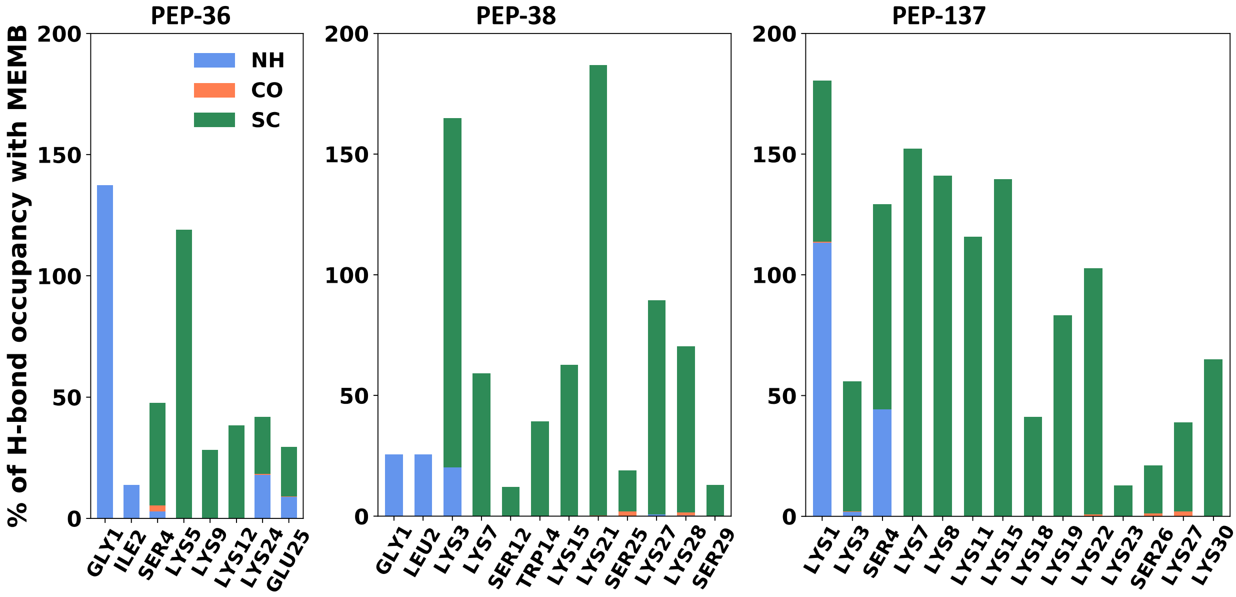

Supplement: Supplementary file 1 [file antibiotics-11-00411-s001.zip › Supplementary Tables and Figures.pdf]
